# Genome assemblies and annotations are not static and need support for tracking their evolution

**DOI:** 10.1093/bib/bbag357

**Published:** 2026-07-03

**Authors:** Nicholas J Dimonaco, Amanda Clare, Martin Vickers

**Affiliations:** Institute for Global Food Security, School of Biological Sciences, Queen’s University Belfast, 19 Chlorine Gardens, BT9 5DL Belfast, United Kingdom; Department of Computer Science, Aberystwyth University, Llandinam Bldg, Penglais, SY23 3DB Aberystwyth, United Kingdom; Department of Computer Science, Aberystwyth University, Llandinam Bldg, Penglais, SY23 3DB Aberystwyth, United Kingdom; John Innes Centre, Norwich Research Park, Colney Ln, NR4 7UH, United Kingdom

**Keywords:** genomics, annotations, version control, file formats

## Abstract

For over 25 years, genomic data have been distributed in two key file formats: FASTA and GFF. These formats are used in nearly all genomic analyses and encode both genomic sequence data and the positions of annotated features. Long-read sequencing, chromatin conformation capture, and advanced assembly algorithms now enable chromosome-level assemblies and pangenomes even for the largest eukaryotic genomes. Genome consortia routinely update assemblies and re-annotate gene models as methods and knowledge improve, yet these updates occur without systematic documentation of what has been modified. As genomics enters the next era, the lack of systematic versioning becomes limiting: different annotation versions cannot be computationally compared, algorithmic improvements are invisible to downstream users, and accumulated biological knowledge exists only in human-readable documentation disconnected from the data itself. Conventional flat-file formats lack the structure to reflect this evolving landscape. While software engineering solved analogous challenges with version control decades ago, for genomics, version control is left to researchers to organise with filenames, directories, or README files. This approach cannot scale to the continuous generation and improvement of millions of genomes. We examine the limitations of genome file formats, demonstrate why incremental improvements are insufficient, and argue that genomics must adopt version control with the same gusto that is applied to generating new sequencing data. Drawing on lessons from software engineering, we outline requirements for better scientific collaboration, machine-readable formats that can capture changes, maintain complete provenance, and enable the reproducible, large-scale biology that the next 25 years demands.

## Introduction

The last 25 years have seen a profound transformation of genomics that began with the first draft human genome at a cost of *∼ *$3 billion using Sanger sequencing [1]. Long-read technologies such as PacBio HiFi [2] and ultra-long Oxford Nanopore [3], coupled with chromatin interaction sequencing such as Hi-C [4] or Omni-C [5], now enable eukaryote chromosome-level assemblies to be built for <$35 000, a reduction of *∼ *100 000*× * [6]. The scale of this transformation is reflected in the non-redundant RefSeq database [7], which has experienced a ~290*× * increase in unique accessions, and the public repository GenBank [8], which has grown ~813*× * in community-deposited sequences.

As sequencing technologies, assembly pipelines, and curated biological knowledge evolve, many assemblies and annotations are periodically updated to improve accuracy. Although standardisation initiatives exist, such as the NCBI Prokaryotic Genome Annotation Pipeline (PGAP) [9], which is used to reannotate prokaryotic genomes ‘once a year on a rolling schedule to incorporate annotation updates due to software or evidence improvements’ [10], the pipeline itself has undergone substantial changes in its annotation tools and evidence models. These revisions are not systematically documented in ways that allow downstream users to trace differences between releases or reconstruct how annotations have evolved over time. Similar challenges have been observed in large-scale reference sequence databases [11] and genome curation efforts, where iterative assembly refinement and reannotation, particularly following the integration of long-read sequencing technologies, often require repeated manual comparison between releases without formalised systems for tracking, summarising, or contextualising those changes [12]. Custom infrastructure that used file paths under version control and dedicated tracking databases was required to manage the hundreds of breaks, joins, and corrections applied during manual curation. The challenges associated with tracking annotation evolution are not restricted to newly generated or poorly characterised genomes. Even major reference resources for model organisms undergo substantial revision. For example, the transition from the mouse reference assembly GRCm38 to GRCm39 represented the first coordinate-changing update to the mouse genome in 8 years, requiring extensive remapping of annotations and associated biological metadata by resources such as Mouse Genome Informatics (MGI) [13]. Given the central role of the mouse model in genetics, genomics, and biomedical research, such changes propagate through a vast ecosystem of databases, analytical workflows, and published studies. Without mechanisms to explicitly record and summarise annotation differences between releases, researchers are left to infer how updates may affect biological interpretation, reproducibility, and comparisons with previous work. Independent initiatives such as AllTheBacteria [14] provide greater consistency across large genome collections, yet they similarly lack mechanisms to directly compare or summarise successive assembly and annotation versions. For example, assemblies have already undergone retrospective modification across releases, including the removal of human-contaminated contigs and the replacement of earlier datasets, with users required to combine base and incremental releases to reconstruct a complete dataset. These data are distributed across multiple releases and file structures, often without explicit linkage between corresponding assembly and annotation versions, leaving users to infer which files are compatible and what has changed between releases. As a result, researchers lack a clear and reliable means to assess how version-to-version changes affect biological interpretation.

For prokaryotic gene-centric pangenomics in particular, the lack of systematic tracking of assembly and annotation changes presents a major obstacle. Comparisons across thousands of genomes can be derailed by undocumented updates, leading analytical tools to interpret technical artefacts as biological differences and thereby distorting pangenome size, composition, and evolutionary inference. With the emergence of affordable long-read sequencing, whole-genome plant pangenome projects have expanded rapidly [15–17]. Multiple laboratories now contribute to produce assemblies and downstream resources such as repeat masks, transposon annotations, and gene models [18]. As chromosome-complete assemblies become routine and teams work in parallel, coordinating updates across groups risks becoming a significant organisational bottleneck. This coordination cannot be achieved through improved file naming conventions, expanded directory structures, or longer README files and meeting notes alone. Even at small scales, researchers frequently encounter FASTA and GFF files in which the sequence identifiers in the FASTA headers do not match those in column 1 of the GFF, with no clear way to determine how or why the discrepancy arose.

This situation requires more than incremental adjustments; it demands a comprehensive rethinking of how genomic data are represented and managed. Such a shift must encompass not only file formats capable of capturing richer metadata but also integrated version control and provenance tracking systems that trace the evolution of genomic data across technical, computational, and interpretive dimensions. Currently, researchers have no meaningful way to determine what has changed between releases and thus may select outdated versions without being able to detect whether their selection has study-defining consequences. Just as software development was transformed by distributed version control systems that enabled collaboration, reproducibility, and systematic change tracking, genomics now requires analogous tools to manage dynamic biological datasets. Genomic data must evolve from static snapshots into living, traceable repositories that preserve the history of scientific discovery and technical refinement, thereby strengthening comparative analysis and reproducibility. We highlight the need to transform genomic data from static snapshots into living, traceable repositories that preserve the full history of scientific discovery and technical advancement, ultimately enabling more robust comparative analyses and ensuring reproducibility.

## Limitations of current genomic data formats

While the simplicity of the FASTA [19] and GFF [20] file formats has been key to their wide adoption and interoperability across tools and workflows, it also limits their ability to support the modern requirements of genomic data management. These formats often fail to capture critical contextual information. Although genome assemblies and annotations are routinely revised and improved, users typically see only the ‘final’ FASTA or GFF file, stripped of the rich context that would explain why and how it differs from earlier versions. This is limiting, particularly in large comparative genomic or pangenomic studies, where subtle differences can cascade into incorrect biological conclusions.

The following sections detail the challenges associated with the use of FASTA, GFF, and other genomic data file formats. There have been numerous versions of the GFF and GTF [21] file formats, and while each have their differences, where mentioned, ‘GFF’ is extensively used to refer to version 3 (GFF3) as it has become the standard for almost all contemporary workflows.

### Sequences, their metadata, and the versioning challenge

Many domains use systems that track changes to data over time, record what was altered, preserve previous versions, and distinguish minor adjustments from major restructurings. These systems assume the underlying data are structured to allow meaningful comparisons between versions. Genomic formats do not satisfy these assumptions.

FASTA has become the *de facto* format for distributing genome assemblies, transcriptomes, and large DNA/protein datasets, despite originating as a minimal sequence container described only briefly as an aside in an academic paper and lacking formal governance [19]. The name itself derives from the FAST family of sequence similarity search algorithms. FASTP was originally developed for protein searches and was significantly faster than the earlier SEQHP (Goad–Kanehisa) method, which its authors described as being merely ‘satisfactory for random sequences’ [22, 23]. Subsequent publications introduced FASTN for nucleotide searches and FASTA, where the ‘A’ is generally interpreted as ‘All’, indicating support for both protein and nucleotide sequence comparisons [24]. However, while the FASTA algorithm became widely adopted, the associated sequence text representation evolved informally into what is now known as the FASTA file format, without ever being formally standardised or independently specified. Ironically, although the algorithms themselves are open source [25], access to the original 1985 journal article remains behind a paywall [19].

While FASTA simply represents a genome’s state at a given point, it records neither how that state was reached nor its supporting evidence. It is simple, flat, plain-text structure, in which sequences are introduced by header lines beginning with ‘>’, provides no standardised mechanism for embedding structured metadata. Current version control systems (discussed in Section Lessons from software engineering) assume code or free text with natural break points (in the form of words) and structure (in the form of functions, modules, sentences, and paragraphs). As a result, contextual information is forced into *ad hoc* headers or inferred from file extensions (e.g. .fna, .faa), leading to fragmented or externally dependent documentation. As is often the case in bioinformatics, the precise reasons for FASTA’s adoption as a *de facto* standard are difficult to reconstruct and likely reflect a mixture of convenience, timing, and community uptake rather than formal design.

The graphical fragment assembly (GFA) format [26] addresses FASTA’s main limitation by preserving not just assembled sequences but also their relationships and uncertainties. Rather than reporting contiguous segments of assembled sequences, known as contigs, as isolated strings, GFA represents genomes as graphs where nodes are sequence segments and edges record connections or overlaps. This captures repeats, alternative paths, and unresolved haplotypes typically collapsed into single consensus sequences in FASTA files. By retaining the evidence and structure behind each join, GFA makes assemblies easier to compare, update, and interpret, since changes affect specific graph elements instead of replacing entire multi-million-base contigs. Designed to capture alternative paths, structural variation, and assembly uncertainty [27], it enables programmatic traversal of contig, bubble, and haplotype branches. GFA files can export simplified FASTA representations by walking graph paths, while retaining fuller complexity. However, metadata remains sparsely defined, and large assemblies quickly produce files that are difficult to validate, compare, or integrate without substantial auxiliary tooling. GFA represents a potential basis for a ‘genomically aware’ version control system exploiting graph-based structures.

### Genome annotations, their metadata, and the versioning challenge

Genomic annotation formats such as GFF face analogous limitations to the sequence formats. As genome projects have matured, annotations have become richer, more complex, and subject to frequent updates. Yet GFF’s flat structure provides no standardised way to record annotation provenance, document changes across versions, or describe which tool, database, or evidence supported specific gene models. Crucially, beyond the uncontrolled ‘source’ or ‘attribute’ columns, the format itself cannot document why features change, whether annotations were computationally predicted or manually curated, or how versions relate to each other.

Despite the specific goal of the third version of the GFF format (GFF3) to provide ‘universal’ standardisation by incorporating “the most common extensions to GFF, while preserving backward compatibility with previous formats” [20], practical usage depends heavily on user interpretation. While many annotation tools reportedly conform to the GFF standard, each commonly requires bespoke format handling due to often obscure and subtle differences [28]. This informality is reflected even in the evolution of the format's name, which changed from Gene Finding Format [29] to the current General Feature Format.

### Interpreting changes in genomics data

When genome assemblies or annotations are updated, downstream analyses built on previous versions may become obsolete or misleading, yet without clear documentation of changes, researchers cannot assess the impact of these updates on their work. The problems with applying version control to FASTA and GFF extend beyond simple provenance tracking to fundamental issues with how changes are represented and interpreted.

The coordinate-based nature of genomic annotations creates critical complications. For example, when gene models are refined (such as moving exon boundaries, merging adjacent genes, or splitting a single gene into multiple isoforms), contemporary diff algorithms treat these as wholesale feature replacements rather than evolutionary changes to the same biological entity. An exon added to an existing transcript or a pseudogene reannotated as a functional gene through experimental work appears as an entirely new feature rather than structural or evidential modifications. Additionally, assembly updates (splits, merges, and reorientations) shift all genomic coordinates, causing every annotation line to appear ‘changed’ despite the biological annotations remaining identical relative to genomic context. Gene identifier reassignments compound these problems. A gene labelled ‘gene001’ in one version might become ‘gene042’ in the next for a variety of reasons, such as the software used or arbitrary feature-ordering numbering. Version control systems interpret this as, at best, a rename, or at worst, deletion of one gene and the creation of an entirely unrelated gene, despite the underlying biological feature remaining essentially unchanged. We discuss this further in Section Transferring annotations between assemblies.

Furthermore, GFF’s unenforced hierarchical relationships prevent version control systems from meaningfully accessing update differences. These limitations will increasingly hinder genomic data users as sequencing technologies and bioinformatic methods advance.

### The informality paradox

Informal file formats such as FASTA and GFF, with their simplicity, flexibility, and lack of rigid structure, have been incredibly successful at enabling researchers with limited computational experience to develop powerful tools that advance biological understanding. Biology is complex, and to model it, simplifications and compromises have had to be made. The result is a paradox where the very features that made them successful effectively render them incompatible with the systematic change tracking that modern collaborative genomics needs.

## Lessons from software engineering

The challenges facing genomics in data management, version control, and reproducibility are not unique to biology. Several other fields have successfully addressed similar problems through the development of sophisticated data management frameworks and version control systems. Perhaps the most instructive example comes from software engineering, where the development of distributed version control systems such as Git [30] and Mercurial [31] has revolutionised collaborative software development. Software version control systems offer several features that would be transformative if adapted to genomics: atomic commits that bundle related changes together, branching and merging capabilities that allow parallel development tracks, comprehensive change logs that document the rationale for each modification, and distributed repositories that enable decentralised collaboration while maintaining data integrity.

### Change identification and ownership of changes

Aggregating coherent modifications into commits with documented rationales and contributor identities is essential to version control. Each commit receives a unique identifier serving as a milestone, enabling researchers to revert to previous versions or determine whether others have adopted specific changes. Documenting contributors encourages broader participation beyond single maintainers, attributes credit appropriately, and provides contacts for inquiries about specific modifications.

### Semantic versioning and incremental releases

Semantic versioning, widely adopted in software development, provides a standardised framework for communicating the significance of changes through version numbers. This system distinguishes between major changes (x.0.0) that break compatibility, minor changes (0.x.0) adding functionality, and patch-level (0.0.x) bug fixes. A similar framework could signal to genomic researchers whether updates require re-analysis or simply offer additional features.

The human genome reference GRCh38, released by the Genome Reference Consortium (GRC) in December 2013 [32], exemplifies both the value and limitations of genomic versioning. Fourteen non-coordinate-changing patches have since been released, reserving major number changes for coordinate-breaking updates. However, patches can contain substantial modifications. GRCh38.p14 included 69 new scaffolds with inversion haplotypes, new gene/allele representations, sequence extensions and corrections to component order [33]. The fact that the major number remains unchanged for over 12 years [32] indicates a reluctance to release further disruptive changes. GRCh39 is now indefinitely postponed. Meanwhile, the Telemore-to-Telomere and Human Pangenome Reference Consortia have released alternative versions of the human genome [34, 35], creating reference inconsistencies that complicate change tracking and demonstrably affect exome variation studies [36].

Version management systems enable users to adopt updates at their own pace while tracking progress and anticipating future developments. Time-based release strategies are common, and the benefits of frequent scheduled releases are well understood [37].

### Merging conflicting changes

The collaborative nature of science depends on identifying and correcting errors uncovered during peer work, yet resolving conflicting edits to genomic data remains slow and largely manual. For example, as assemblers output contigs in arbitrary order and orientation, curators must manually impose chromosome naming, ordering, and orientation, often guided by previous references. For large genomes such as plants, visually reorienting contigs into chromosomes is still routine [38]. When different assembly tools are applied to the same data, harmonising divergent outputs becomes laborious and error-prone.

Conflict resolution will never be trivial in a domain as dynamic as genomics, but other fields offer useful models. Software engineering tools like Git automate merges where possible and expose ‘detailed diffs’ where human judgement is required, supporting distributed editing without central control. In contrast, online editors such as Google Docs or Microsoft Word rely on timestamped keystrokes and Operational Transformations [39] to merge edits automatically, but require a central server to coordinate changes. Choosing between decentralised, human-directed merging and fully automated, centralised systems involves real trade-offs. Emerging work on conflict-free replicated data types suggests a future in which independently edited genomic repositories can merge deterministically [40], though only recently have such approaches become efficient enough to be practical.

### Working in teams

The working practices of software engineers have been transformed by version control systems, enabling teams to work asynchronously across locations, time zones, and files while still merging updates when needed. Synchronous editing systems such as Google Docs, Overleaf, and Microsoft Word similarly allow multiple people to edit the same document in real time, supporting fast collaboration, immediate visibility of changes, and a shared understanding of the current state of the work.

Version-controlled systems can be distributed or centralised. Distributed systems let each team member keep their own copy and full history, work independently, and later resynchronise changes, resolving conflicts manually. This approach provides built-in redundancy and allows progress without waiting for others. Centralised systems maintain a single authoritative version and rely on automatic conflict-resolution algorithms to keep it consistent, but they introduce a single point of failure and may force users to delay updates to avoid disruptive merges. Regardless of the model, clearly defining who can modify the data and when remains essential for effective teamwork.

### Descriptions of differences

Tools like diff [41] and diff3 [42, 43] illustrate how version control systems can precisely identify both historical and prospective changes between data states. Software version control provides line- and character-based difference reports showing precisely what changed and where. Determining the differences (especially those that may break or change genomic features) that any new patch would make to a current copy of the data would also be valuable. However, these tools provide technical descriptions of differences, not semantic ones and cannot indicate whether a coordinate shift represents biological refinement or an assembly artefact. Even in public-facing systems such as Wikipedia, users are limited to seeing character-based displays of insertions and deletions when comparing two versions from history.

### Community and forges

Software version control systems often integrate with ‘forges’; sites that provide additional support for the community, such as issue and bug reporting and tracking, documentation, email lists or forums, and other tooling to help users and developers work with the changes. Sourceforge, GitHub, GitLab, and BitBucket are or have been examples of forges for software engineering repositories.

Institutions such as Ensembl and NCBI provide single points of access for vast collections of genomic data; however, this can be fragmented with various sources and versions made available that are difficult to compare. Genomic forges, with provenance and version tracking, could provide some level of support to users for both genomic data distribution and use.

Forges encourage community standards for submissions, such as continuous integration tests and style guides for coding, like Python’s PEP8 [44]. Genomics would benefit from similar standards for scaffold naming, GFF adherence, and hierarchical representation of genetic elements. Forges also encourage the community to provide unambiguous licenses for the use of the resources and to provide discussion forums to raise and track issues and follow the comments of other experts and users.

### Example inspired by software engineering

**Figure 1:**
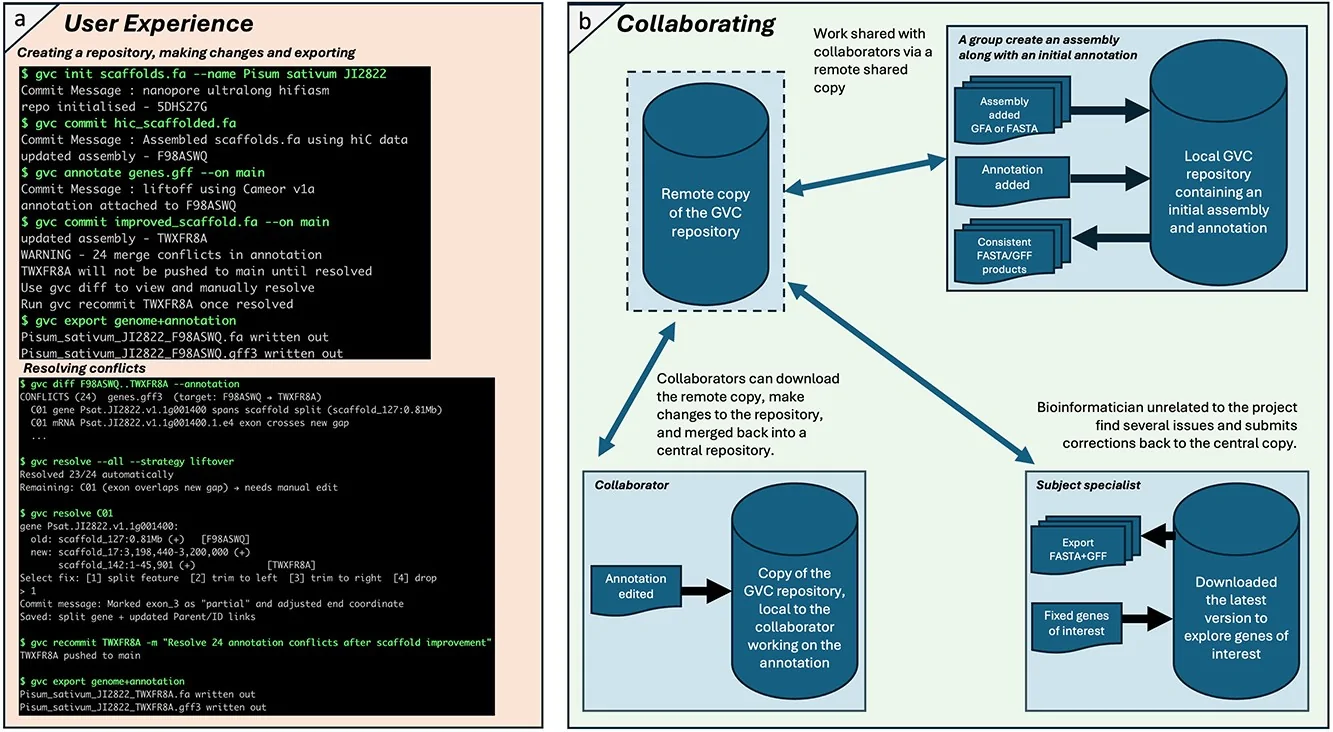
How might one use a git-like version control system for genomics? (a) Left: How a fictional command line ‘genome version control’ program might appear to the user, adding/exporting data, committing changes along with resolving conflicts (b) Right: A simplified view of how collaborators could share and work together on a version-controlled genome.

Figure 1 provides an example of how genome version control might look if it were similar to the systems used in software engineering.

This figure also provides a scenario. A repository is initialised and populated with a pea genome assembly. The assembly is improved by adding hiC data and then committed. An annotation is made by lifting over features from another similar genome, and this is added to the repository. The assembly is improved further, but this time the commit raises conflicts in 24 of the gene annotations. Most of these span a scaffold split and can be resolved automatically. The remaining one requires a manual decision, and after inspection, the user chooses to split the exon before the changes are committed.

However, versioning systems similar to those in software engineering are not the only possibilities. The needs of genomics are likely to be very different to the needs of software.

## What new features are needed for the future of genomics data versioning?

In addition to these lessons learnt from software engineering, the following specific features and requirements will be necessary in order to envision a comprehensive solution for modern genomic data management.

### Outputs must be compatible with existing genomic file formats

Rich metadata support could capture not only sequence information but also contextual data describing sample provenance, analytical methods, computational environments, and decision rationales. Such metadata may be structured using controlled vocabularies and standardised ontologies to ensure machine readability and interoperability across platforms and institutions. Any new system should provide clear migration paths from existing formats while remaining flexible enough to accommodate future technological and analytical developments. At the same time, outputs in established formats such as FASTA and GFF will continue to be required for compatibility with current tools and workflows.

While machine readability is essential for automated pipelines and quality control, versioned outputs must remain immediately recognisable to data owners and users. Processing should not introduce superficial syntactic changes that generate widespread file differences without substantive biological meaning, such as arbitrary formatting shifts or unnecessary renaming of genes. A versioning framework will, therefore, preserve semantic intent and minimise cosmetic alterations, ensuring that updates clarify rather than obscure the curators’ original decisions.

### The assembly and annotation are linked by coordinates

Annotation files will likely need to be coordinate-dependent representations of the underlying assembly; any change to one, therefore, has consequences for the other. In simple cases, insertions or deletions require systematic coordinate adjustment. More complex reassemblies (such as the movement or inversion of a genomic segment containing annotations) could result in annotation updates that explicitly record those structural transformations rather than representing them as artificial deletion and reinsertion events. Achieving this will likely require an underlying data structure that integrates assembly and annotation, ensuring coordinated updates while still permitting the export of standard individual files through appropriate tooling.

### Updates may be applied in different orders

Updates to biological data often originate from multiple research groups working on distinct genomic regions or functional domains. One group may refine alternative splicing annotations while another improves centromeric assemblies, and these contributions may be submitted independently. Users may wish to incorporate (pull down) specific updates into their local datasets without necessarily adopting all concurrent changes. Traditional software version control systems typically organise history around a primary branch with auxiliary branches that are eventually merged. Biological data versioning, however, may require greater flexibility. Patch-based systems such as Darcs and Pijul [45–47] model changes as composable patches that can, subject to defined dependencies, be applied independently or in alternative orders. These approaches explicitly consider commutativity, associativity, and dependency relations between updates. Enabling selective application, reversal, and reordering of biologically meaningful updates would allow genomic scientists to integrate new knowledge in a controlled and transparent manner as understanding evolves.

### Biologically relevant diffs

Sophisticated tooling could generate biologically meaningful diffs that describe not only what has changed between versions, but also the functional consequences of those changes. Rather than relying on character-level or line-based comparisons, such systems could summarise which chromosomes or regions are affected, whether annotations have been added, removed, or modified, and whether sequence alterations intersect previously annotated features.

These capabilities would support comparisons not only between successive versions, but also across branches, between genomes within a pangenome, or between alternative assembly and annotation pipelines applied to the same underlying data.

### Recording differences of opinion and alternative branches

Large genome and pangenome projects often involve multiple research groups, each generating annotations, predictions, and interpretations that must be reconciled into a single, coherent resource. A version-controlled system offers a way to record, compare, and manage these differing viewpoints. Branches can preserve alternative annotations arising from different tools, pipeline versions, or expert judgements: e.g. competing gene models, uncertain start codon positions, or alternative splicing possibilities. Rather than forcing an early choice, these branches allow the community to retain all plausible interpretations while designating a primary model for current analyses. Functionally, this is similar to viewing multiple annotation ‘tracks’ in genome browsers, but with the added advantages of version tracking, reproducibility, and clear histories of how disagreements were resolved.

Software such as Mikado [48] could be used to integrate multiple annotations from multiple branches if a single GFF file is needed, while the individual alternatives remain in branches in the repository until future evidence can be found to resolve the alternatives. Although some aspects of annotation reconciliation can be automated, many decisions will continue to rely on expert discussion and community consensus. A structured branching workflow ensures that these decisions are transparent, auditable, and reversible. These features would be increasingly indispensable for large-scale genome and pangenome projects.

### Performance

Scalability considerations are important as genomic datasets continue to grow exponentially. Future formats must handle not just larger individual datasets but also the complex relationships between datasets in pangenomic and population-scale studies. This requires efficient storage mechanisms, intelligent indexing strategies, and support for distributed data management.

Software version control systems such as Git assume that file sizes are small and that saving full file snapshots is a worthy trade-off for the time it would take to compute and save only the differences. For genomics data, however, clearly, if we change only one character in a 2 Gb FASTA file, a version control system that re-uploads the entire file will be inefficient and could mean that calculating and saving patches instead becomes cost-effective. A scalable system for genomic data needs to be efficient locally rather than slow to rebase centrally.

### Evidence-based annotation quality and confidence metrics

Version control tracks when annotations change, and what changed, but scientific value depends equally on knowing how confident we should be in each annotation. Annotation stability correlates only loosely with accuracy. An unchanged decade-old gene model may simply reflect that nobody re-examined it, while a newly added annotation based on experimental evidence may be far more reliable. A comprehensive genomic data system could capture both version history and evidence quality. For sequences, this means labelling regions of lower quality, repeat arrays of uncertain length, gaps with estimated sizes, and segments where assembly algorithms made ambiguous choices. For annotations, it means distinguishing gene models supported by direct experimental evidence (mass spectrometry confirming start codons, long-read RNA-seq supporting complete exon structures) from purely computational predictions. Some precedents exist: UniProt/Swiss-Prot [49] includes detailed peptide mapping data, RefSeq [10] annotations include ‘experiment’ and ‘model_evidence’ attributes for some organisms, and the evidence and conclusion ontology (ECO) [50] provides controlled vocabularies for evidence types. However, GFF and other standard formats lack agreed conventions for reporting evidence systematically, leaving information in *ad hoc* attribute fields that preclude automated integration. Multiple alternative annotations with different evidence profiles could coexist in a single version: a gene with three proposed start codons (one proteomics-supported, one from homology, one *ab initio*). Rather than forcing premature choices, version-controlled systems could maintain alternatives, allowing users to select annotations meeting their confidence thresholds. This differs from software branching: genomic alternatives may persist indefinitely, representing genuine biological uncertainty rather than temporary workflow divergence. As new evidence accumulates, annotations gain improved evidence scores alongside version updates, creating an auditable record of how biological knowledge evolved.

## What solutions are there for non-static evolving genomic data?

The way forward for genomic data will demand new ideas combined with existing technology. Here we discuss prior work towards version control for biological data, high-performance genomic file formats that could enable versioning and diffing, and existing methods for recording, tracking, and describing changes to sequence and annotations.

### Previous work towards version control for biological data

While version control for genomic data files remains largely unsolved, several initiatives have attempted to address broader data management challenges in bioinformatics and computational science. These efforts, though not specifically designed for genomic sequence files, provide valuable insights.

#### Pipeline-focused data versioning and workflow provenance

Systems such as Pachyderm [51] and RO-Crate [52] aim to improve version control and provenance tracking in scientific workflows. Pachyderm applies Git-like methods to biological data pipelines, enabling automatic data processing upon updates, beneficial in bioinformatics for data lineage and immutability. RO-Crate (Research Object Crate), aligned with the W3C PROV provenance standard [53], captures workflow execution and provenance, bundling associated objects and their metadata. Both systems highlight provenance tracking’s importance in computational biology but face challenges with genomic data. Pachyderm’s reliance on Kubernetes [54] and opaque file handling limits its effectiveness, while RO-Crate cannot track detailed file version changes, only documenting input–output relations. These systems track workflows and data lineage but fall short in versioning file contents.

#### Committing large data files

DataLad [55] manages large scientific data by using Git and git-annex (https://git-annex.branchable.com/). It uses git-annex to manage file content separately from Git, thus tracking large data files without the performance issues of Git. Instead, the content is held in the annex, and a checksum of the file’s content is committed. Commit messages can be automatically generated to record the command invoked to produce the data. The use of checksums for ensuring correct download of large genomics files is already widespread. Committing checksums for version history could offer a limited step towards versioning large files. However, like other Git-based methods, DataLad would treat large or frequently changing genomic files as atomic units, storing new copies with each change, making it unsuitable for fine-grained genome updates.

#### Domain-specific versioning approaches

Beyond general-purpose data management systems, domain-specific efforts have explored version management for particular types of biological data.

CellRepo [56] applies version control principles to synthetic biology, implementing a cloud-based system for tracking engineered cell lines throughout their development. It logs digital data and commits, detailing genotype, phenotype, and more. They propose inserting physical DNA barcodes into cell chromosomes to link living samples back to their digital commit history and engineering decisions. However, CellRepo focuses on metadata about engineering processes, not large genomic files.

VerTIoN [57] addresses the challenge of storing and querying multiple related versions of large datasets, a problem directly analogous to genomic sequence versioning. It uses a tree data structure to efficiently store and query tissue-specific biological networks that share substantial overlap but differ in specific contexts. Ancestors in the tree are network components shared by all descendants, and siblings in the tree represent alternative tissue-specific components of the network. Despite its efficiency for networks, VerTIoN’s model would need substantial adaptation to handle the kinds of changes common in genome assemblies, where sequences may be split, merged, reordered, or corrected rather than simply added.

Gen [58] is a new tool for synthetic biology that is designed to track edits to plasmids and other sequences. It offers a git-like command line tool (gen) and a web interface (GenHub) with issue tracking. The command-line tool provides the ability to commit insertions and deletions, create character-based diff reports, see a graphical view of the current sequence, export FASTA and GFA formats, use a VCF file to update a genome, and propagate annotation changes to a GFF after edits to the sequence. However, the actual tracking of versions is at present still underdeveloped: lineage is represented as introducing new (child) samples, annotations are not stored, and files are not tracked. This tool is perhaps the closest so far to offering the functionality needed for full version control of genomic sequence and annotations.

### High-performance formats that might help build a future system

Given the need to interlink assemblies with annotations while supporting efficient version tracking, new data structures may be required. These may be binary formats from which established formats like FASTA and GFF can be exported, ensuring compatibility with existing infrastructure. Several recent formats demonstrate approaches that could go towards addressing some of the fundamental limitations of FASTA and GFF, particularly in the context of pangenomics and graph-based representations of assemblies.

GFA2 [27] can specify assembly graphs at varying levels of detail, from simple topology to comprehensive multi-alignment of reads, and can represent string graphs at any stage of assembly, from initial overlaps to final resolved contigs with multi-alignments. Building on GFA’s foundation, the reference GFA (rGFA) [59] format extends the basic graph model by adding three additional tags to each segment that indicate its origin, creating a stable coordinate system that preserves the linear reference genome coordinates within the graph structure. This design allows positions to be specified using familiar chromosome coordinates (e.g. ‘chr1:9’) while also representing structural variations as graph branches. Complementing rGFA, the graph alignment format (GAF) [59] provides a tab-delimited format for representing sequence-to-graph alignments as a strict superset of the PAF (Pairwise read mapping format) format [60], using stable coordinates to denote paths through the graph.

For large-scale pangenome applications where storage efficiency becomes critical, the GBZ format [61] addresses the inadequacy of text-based GFA when representing many similar paths by using a GBWT (Graph Burrows–Wheeler Transform) [62] index to store path collections in a more space-efficient manner. With this graph-specific optimisation, GBZ provides better than gzip compression for GFA files with many haplotype paths, enabling human-genome-sized pangenome graph analysis on standard desktop computers.

Parallel developments have also improved the efficiency of traditional annotation file access. GFFx [63] represents a more integrated approach to working with GFF files through a Rust-based toolkit to enable fast random access with minimal overhead. The system uses a collection of indices for direct memory access to enable fast feature extraction, attribute-based searches, and region queries with minimal input/output overhead.

At an even larger scale, MetaGraph [64] demonstrates that it is possible to make petabase-scale sequence repositories efficiently searchable. Using annotated de Bruijn graphs, MetaGraph has successfully indexed 18.8 million unique DNA and RNA sequence sets comprising 67 petabase pairs of raw sequence data. The authors showed that this capability enables large-scale analyses such as surveying antimicrobial resistance genes across hundreds of thousands of human gut microbiome samples and identifying circular RNAs across major transcriptomics cohorts. MetaGraph has made the entire publicly available NCBI Sequence Read Archive fully text searchable.

These graph-based formats and indexing approaches represent a significant conceptual advance over linear file formats like FASTA and GFF. Graph-based assembly formats can natively represent the relationships between multiple genomes or assembly versions within a single coherent data structure, while modern indexing systems like GFFx demonstrate that substantial performance improvements are possible even when working with traditional flat-file formats. However, these innovations remain primarily focused on either assembly graphs and structural variation (GFA, rGFA, GAF, and GBZ) or efficient data access (GFFx and MetaGraph) rather than addressing the broader challenges of annotation provenance, version tracking, and comprehensive metadata management. Nevertheless, their success in encoding complex genomic relationships efficiently and enabling high-performance access suggests that similar structured approaches (whether graph-based, hierarchically indexed, or hybrid systems) could be adapted to solve the version control and metadata problems that currently limit genomic data management.

A table summarising strengths, limitations, version control compatibility, and future potential of genomic file formats, including those discussed in this section, is provided in Supplementary File 1 (Supplementary Table 1).

### Existing methods for describing changes to sequences

Biologically relevant ‘diff’ reports of the changes between versions of a genome sequence and its annotation would help users to understand whether they should migrate to a newer version and how this will affect their downstream analysis. Coordinate-level descriptions of change and higher-level descriptions will need to be combined to give the full picture. Whether the version control system stores full files that need to be compared or just patches that need to be applied, mechanisms for translating the change into interpretable formats will be needed.

The MUMmer system [65] can efficiently determine the character-level similarities and differences between two or more whole genomes by first finding maximal exact matches. It provides a dnadiff tool that outputs the similarities (matching regions) between aligned genomes in a tab-separated format. This software is capable of aligning long eukaryotic genomes. While MUMmer could be used to provide a coordinate-level diff of exact matches, and will report an exact match if a piece of sequence has been moved to another location, it has no internal representation of the units of sequence, or the operations that could represent the changes.

At a character-level, changes to a genome can be denoted as individual insertions, deletions, or substitutions. A higher-level model of changes, the DCJ-Indel model [66], based on an earlier DCJ (double cut and join) model [67], provides a description of the gain and loss of larger sequence elements from plasmids over the course of evolution. It uses cutting and joining operations to handle inversions, translocations, fusions, and excisions. This model has been used to determine evolutionary distance, but measures such as this could form the basis of a sequence diff report for more biological meaning.

### Existing methods for recording changes to annotations

Current approaches to managing genome annotation versions vary widely across major databases and initiatives, reflecting the historical lack of standardised practices for tracking annotation changes. These methods range from sophisticated stable identifier systems to minimal documentation, with most falling short of providing the comprehensive version control that would enable researchers to understand precisely what changed, why, and with what evidence.

#### Stable accession systems for sequences and assemblies: tracking what, not why, or how

For nearly 40 years, the International Nucleotide Sequence Database Collaboration (INSDC) [68], now comprising NLM-NCBI [69], EMBL-EBI [70], and ROIS-NIG [71], established stable accession version identifiers to address reproducibility concerns well before many of the version control challenges discussed in this review became acute. The sequence accession version system, operational since February 1999 [72], provides a standardised mechanism to uniquely identify sequences and their versions. When updated, the accession remains constant while the version number increments (e.g. NC_000001.10 to NC_000001.11), allowing researchers to specify exactly which version was analysed.

Building on this foundation, assembly accessions (GCA_ for GenBank assemblies, GCF_ for RefSeq) were introduced around 2009–2011 to describe coherent sets of sequences representing complete genome assemblies [73]. Assembly accessions similarly use version suffixes (e.g. GCA_000001405.1 for GRCh37 and GCA_000001405.15 for GRCh38) that increment with updates. The INSDC’s commitment to stable identifiers is formalised in their collaborative arrangement, which mandates that members provide ‘mechanisms for submission and access to submitted data, associating a citable accession number to submitted objects’ and ensuring data meets FAIR [74] principles [68].

Databases such as Ensembl [75], WormBase [76], and FlyBase [77] maintain feature-level stable identifiers for genes, transcripts, and proteins that persist across releases unless the underlying models change substantially. When changes do occur, the stable identifier receives a version number increment, allowing researchers to track the evolution of specific features over time. ‘Unlike gene names which can change as a result of improvements in scientific knowledge, stable identifiers should continue to refer to the same genomic features’ [78].

However, these systems have significant limitations. They document *that* a change occurred and provide access to both versions but offer no machine-readable documentation of *what* changed, *why*, or *how* changes affect downstream analyses. When annotations are recomputed for new assemblies or manual curation is merged with automatic annotation, the rationale for changes remains scattered across release notes, genome browser displays, and human-readable documentation that is completely disconnected from the annotation files themselves. A researcher downloading a GFF file has no programmatic way to determine what changed from the previous version or why.

#### RefSeq and the wholesale re-annotation problem

NCBI’s PGAP exemplifies the wholesale re-annotation challenge: when the pipeline is updated, NCBI re-annotates tens of thousands of genomes to maintain consistency. GenBank [79] and RefSeq records include a COMMENT section documenting which pipeline version, annotation method, and software revision were used, but this high-level metadata does not indicate what changed from previous annotations in any machine-readable form. When PGAP 6.8 was released, NCBI noted it ‘perfectly reproduces 98.6% of the protein models’ from version 6.7, with most differences involving ‘small changes in start site selection’ [80]. However, identifying which specific genes in which genomes were affected requires comparing entire annotation files; a process that is neither routine nor systematic, considering the issues discussed in Section Genome annotations, their metadata, and the versioning challenge.

#### The tool diversity and manual curation challenge

The annotation versioning problem is compounded by tool diversity and the predominantly human-driven nature of annotation quality control. Format standardisation alone is insufficient without standardised practices for documenting changes. When a new tool or updated model is run, researchers generate entirely new annotations with no systematic, machine-readable comparison with previous versions beyond basic overlap statistics that must be manually computed. Manual annotators record their decisions in curator notes or free-text fields that are neither standardised nor machine-parseable. When the HAVANA group [81] performs manual curation that is subsequently merged with Ensembl’s automatic annotation, the specific decisions made by human curators are indicated through evidence codes and transcript tags embedded in GFF attributes, but the detailed reasoning behind those decisions exists only in unstructured comments or external documentation. This expert knowledge, representing years of biological understanding, remains effectively invisible and inaccessible to automated analysis.

Apollo [82] offers a partial remedy by enabling collaborative, browser-based genome annotation where curators can edit features in real time, but it still falls short of true version-aware tracking. While it records changes at the level of feature edits, the rationale behind those edits remains largely unstructured, its history logs are not designed for downstream computational comparison of annotation versions, and there is no provision for changes in the genome assembly.

Large-scale uniform annotation efforts such as AllTheBacteria attempt to address consistency through automated pipelines, but face identical re-annotation challenges as methods evolve. Each new release becomes a new dataset requiring labour-intensive manual comparison to understand changes, with no programmatic interface for tracking what was modified.

#### Transferring annotations between assemblies

Improvements in sequencing and assembly have brought us into an era where generating ‘complete genomes’ is increasingly becoming routine [34]. Yet despite this success, genome annotation has failed to keep pace. For eukaryotes in particular, where complex gene structures, multi-exon architecture, and regulatory variation remain difficult for current methods to resolve accurately, producing accurate gene models is still a challenge [83].

Tools such as Liftoff [84] attempt to address this need by transferring annotations between assemblies of the same or closely related species, offering a mechanism to propagate information into future assembly versions. However, without a structured versioning framework, these transfers merely copy forward the limitations and mistakes of the original annotation set. This challenge is amplified by the fact that annotation methods have changed little throughout the ‘Next Generation Sequencing’ era [85]. In effect, inaccuracies are serially inherited rather than corrected. Newer approaches, including LiftOn [86], extend Liftoff to more divergent species through homology-based mapping. Yet even these strategies rely on the validity of the original gene models and the assumptions embedded within them. These ‘lift-over’ tools solve the mechanical problem of carrying annotations to successor assemblies, but not the semantic problem of knowing which annotations are correct, which are uncertain, and how they have changed across versions.

For genomic analyses to be genuinely reliable, annotation transfer cannot simply move features forward across assemblies. It must preserve the meanings and influences of each change, documenting not only what was altered, but also who altered it and why.

#### The critical gap in computer-readability

A consistent pattern emerges across all systems: version numbers and pipeline methods are documented at a high level in human-readable text, but none provide systematic, computer-readable, structured records of what changed at the feature level, why changes were made, or what evidence supported them. When a gene’s start codon is revised, or an open reading frame gains functional annotation, or splice variants are added, or when manual curation overrides automatic prediction, the computational tools parsing current annotation files are unable to track these changes. The consequences are severe as researchers must either ignore annotation version differences entirely, manually compare annotation files using *ad hoc* scripts, or hope that version differences do not materially affect their analyses. Analyses on different annotation versions may reach different conclusions, but researchers have no programmatic way to determine whether differences stem from meaningful biological refinements or technical artefacts of different pipelines.

## The future of genomic file formats in the era of artificial intelligence

Rapid genomic data growth and emerging artificial intelligence (AI) encourage a rethinking of genomic data structures and management. AI’s capability to derive significant biological insights from sequences continues to be realised, yet AI performance relies on data availability and quality [87, 88]. Current genomic formats, designed for simpler times, are inadequate for today’s machine learning needs, which require processing millions of genomes with consistent metadata. The challenges are already apparent in existing efforts to create ‘AI-ready’ genomic datasets for tasks such as gene function prediction, which rely on reconciling inconsistent annotations across databases via systems like clusters of orthologous groups (COG) [89]. Ensembl’s stable identifiers help track genes across releases but remain disconnected from the underlying sequences and functional evidence, requiring complex cross-referencing not suited to large-scale machine learning. Current formats simply cannot provide that context.

Evidence-based quality metrics are particularly critical for AI applications. Training datasets for gene prediction, functional annotation, or variant pathogenicity must distinguish high-confidence examples from uncertain ones to avoid propagating errors. Treating every annotation as equally reliable is likely to produce worse machine learning models than one that weighs features by evidence strength. Consider gene function prediction: training on computationally predicted functions alongside experimentally validated ones without quality labels conflates noise with signal, degrading model performance. As genomics generates training datasets from millions of genomes, the ability to programmatically filter for high-quality examples or weight samples by confidence becomes essential. Entity tracking also allows for efficient re-training of AI models in incremental model development. Standardised evidence reporting should therefore be considered a core requirement for AI-ready formats alongside version tracking and provenance documentation.

Looking forward to the next 25 years of bioinformatics, the scale of the challenge will only intensify. The volume and diversity of genomes, from microbial pangenomes to global human variation, will grow to a point where manual curation alone is impossible. It is almost inevitable that AI will take over functional prediction and comparative genomics at scale, but only if the underlying data are machine-ready. COG and Ensembl, despite their limitations, offer a glimpse of what is needed: consistent functional groupings and persistent entity tracking. The next generation of genomic data must generalise these concepts into full knowledge graphs that capture evolutionary history, variation, expression, and functional uncertainty in a way AI can reason over.

Like historic transitions from flat files to relational and then graph databases enabled new computational frontiers, genomics must now look beyond FASTA and GFF. A coordinated redesign is required; otherwise, AI-powered biology will continue to stand on a foundation built for the past, not the future.

## Responsibility

The success of modern genomics has been inseparable from a commitment to openness. This openness enabled broad community uptake, independent reimplementation, and long-term sustainability.

As genomic data increasingly inform policy, medicine, agriculture, and environmental decision-making, the responsibility to ensure their transparency and interpretability grows accordingly. Future stakeholders, including clinicians, regulators, biosecurity agencies, industry partners, and the public, may rely on genomic resources long after their original creators or annotation pipelines have changed or moved on. These stakeholders also represent a powerful lever for change and are uniquely positioned to influence the adoption of improved data standards by demanding transparency, version traceability, and reproducibility as prerequisites for use.

The use of version control cannot be enforced on the community, and there would be no clear authority to do so. Instead, if good tools are built, people will use them because of the benefits for themselves and for the quality of their data. In the future, peer pressure may encourage people to use these tools, just as we see in the software engineering community, where coding without version control is seen as poor practice.

Here, we aim to raise awareness among the community of bioinformaticians to think about the problems they have, and to more publicly offer the tools and work practices they are already developing or using towards this end.

## Discussion

We challenge the bioinformatics community to begin building the solutions for this problem. The limitations of current genomic file formats for tracking the evolution of genome assemblies and annotations are not merely inconveniences. They represent fundamental obstacles that undermine reproducibility, obscure scientific progress, and create barriers to the ‘AI-ready’ future of biological research. FASTA and GFF, designed for an era when assembling and annotating a single genome was a monumental achievement, cannot capture the history of annotation decisions, the rationale for assembly refinements, or the evidence-supported functional assignments. The result is a field where researchers routinely work with different versions of ostensibly the same data, unable to determine whether analytical differences stem from meaningful biological insights or arbitrary technical artefacts. The irony is palpable; while tireless efforts have been made to ensure that genomic data can be processed efficiently by computers (and the simplicity of formats like FASTA has indeed made computational techniques more tractable), the very success of these approaches has not only highlighted their fundamental limitations, but also has become barriers to the future of genomics research.

There is a common thread across the successes and challenges we have discussed in this report. Our management infrastructure of genomic data must evolve alongside analytical capabilities. Sustainable growth necessitates formats designed for the scale and complexity we face now, not the problems we faced 25 years ago. The infrastructure must capture temporal dynamics, tracking not just static snapshots but the evolutionary trajectories of our understanding of genomes and the information they contain. Furthermore, we must plan to support the scale of genomic data. Millions of assemblies, billions of annotations, petabases of sequences will require efficient compression, intelligent indexing, and distributed access patterns that make version control practical rather than difficult. What genomics needs is a new hybrid format that bridges the gap between the rich metadata capabilities of modern structured formats like GFA and the accessibility and ubiquity of FASTA and GFF. An interrogatable container designed specifically for genomic data that holds multiple versions of assemblies, annotations, and metadata within a single queryable entity. Users could request specific genome versions, extract particular features, compare annotations across releases, or export to ‘legacy’ formats for compatibility with existing tools, all while the system maintains complete genomic context, tracks all modifications, and enables semantic diffs that understand biological meaning rather than just text changes. The technical foundations exist. Graph databases, version trees, succinct data structures, and semantic web technologies are potential foundations that genomics has yet to synthesise into a cohesive standard.

The challenge facing the genomics community is both technical and societal: can we design formats and tools that meet future needs while ensuring adoption across diverse users, including database providers, tool developers, and most importantly, end users? Biologists, bioinformaticians, database curators, and funding agencies must prioritise data infrastructure with the same vigour applied to data generation. But it is also an open challenge to the community: can we collectively move beyond the comfortable familiarity of current formats and embrace the complexity required to manage genomic data at the scale and sophistication that modern biology demands? The next 25 years of genomics will be defined not just by how much data we generate, but by how effectively we can maintain it, query it, integrate it, and learn from it. The tools we build today will either enable or constrain the biological discoveries of tomorrow.

**Key Points**

- The Problem: FASTA, GFF and other genomic formats, designed over 25 years ago for single genomes, cannot track changes between versions of assemblies and annotations. Researchers routinely work with different versions of ‘the same’ genome without knowing what changed, why, or whether analytical differences stem from biological insights or technical artefacts, creating a reproducibility crisis.
- Why Current Approaches Fail: Traditional version control systems (like Git) treat genomic files as opaque blobs and use line-based diffs that are blind to biological meaning. They cannot distinguish a sequence rearrangement from a deletion+insertion, or recognise when coordinates shift due to assembly updates versus actual biological changes. Major databases (Ensembl, NCBI RefSeq) track version numbers but provide no machine-readable documentation of what changed at the feature level.
- Lessons from Software Engineering: Distributed version control (Git), semantic versioning, branching/merging, and comprehensive change logs transformed software development. Genomics needs similar infrastructure adapted for biological data: understanding that sequences can be split/merged/reordered, that annotations have hierarchical relationships, and that changes need biological semantics (not just character diffs) to be meaningful.
- The Path Forward: Genomics requires a new hybrid format; an interrogatable container holding multiple versions of assemblies, annotations, and metadata with complete provenance tracking, enabling users to query specific versions, compare changes semantically, and export to legacy formats. This is critical not just for reproducibility but for AI-ready genomics, where models trained on millions of genomes need consistent, structured, version-aware data rather than scattered snapshots.

## Supplementary Material

Supplementary_File_1_bbag357

## Data Availability

No data were produced or used as part of this manuscript.
