# Supplementary material for: Genome assemblies and annotations are not static and need support for tracking their evolution
Source: Brief Bioinform. 2026 Jul 3;27(4):bbag357. doi: 10.1093/bib/bbag357 (PMC13331350; doi:10.1093/bib/bbag357)
Supplement: Supplementary_File_1_bbag357 [file supplementary_file_1_bbag357.pdf]

Supplementary Material

Table S1: Comparison of genomic file formats for sequence and annotation data management and potential version control

Format	Primary Use	Strengths	Limitations	Version Control Compatibility	Future Potential
**FASTA**	Sequence storage (genomes, transcriptomes, proteins)	• Universal adoption and tool support<br>• Simple plain-text structure<br>• Human-readable<br>• Minimal computational overhead<br>• Compatible with nearly all bioinformatic workflows	• No standardised metadata mechanism<br>• Ad-hoc header formats fragment documentation<br>• Arbitrary line wrapping creates misleading diffs<br>• Cannot represent assembly uncertainty or alternatives<br>• No formal governance or specification<br>• Traditional version control produces meaningless character-level diffs	Poor: Line-based diff algorithms confused by formatting changes; sequence rearrangements appear as deletions+insertions rather than relocations	Limited unless replaced by structured alternatives; could serve as export format from version-aware systems; simplicity ensures continued legacy use
**FASTQ**	Raw sequencing reads with base quality scores	• Standard format for raw NGS data<br>• Simple four-line structure per read<br>• Includes per-base quality scores (Phred)<br>• Human-readable text format<br>• Universal tool support	• No standardised metadata for experimental provenance<br>• Quality encoding varies (Phred+33, Phred+64)<br>• Ad-hoc naming conventions for paired reads<br>• No formal specification or governance<br>• Cannot represent read relationships or molecular barcodes efficiently	N/A: Raw data format, not versioned entities; however, read processing pipelines and quality filtering thresholds do evolve	Stable as raw data format; metadata improvements needed for provenance (sample info, library prep, sequencing platform); emerging formats (uBAM) provide richer metadata

Continued on next page

Format	Primary Use	Strengths	Limitations	Version Control Compatibility	Future Potential
**GFF3**	Genome annotation (genes, transcripts, regulatory features)	• Tab-delimited structure enables basic parsing<br>• Hierarchical feature relationships via ID/Parent attributes<br>• Widely adopted as annotation standard<br>• Human-readable coordinate-based representation	• No standardised provenance or change documentation<br>• Coordinate-based nature: all lines change when assemblies update<br>• Heavy user interpretation despite "universal" standardisation goal<br>• Cannot distinguish biological changes from technical reformatting<br>• Flat structure provides no audit trail<br>• Hierarchical relationships not strictly enforced	Very poor: Coordinate shifts cause every annotation line to appear modified; gene ID reassignments interpreted as deletions+creations; format inconsistencies highlight trivial differences while missing substantive changes	Could serve as export format; requires replacement with a structured system capturing annotation evolution, evidence codes, and decision rationales for AI-ready genomics
**GFFx**	High-performance GFF indexing (not a format itself, but a toolkit)	• 10-80x faster ID-based extraction than existing tools<br>• 20-60x faster region retrieval<br>• Model-aware indexing system<br>• Memory-mapped I/O<br>• Low memory use and small index size<br>• Multiple complementary lightweight indices	• Toolkit rather than format standard<br>• Optimises access to existing GFF, not versioning<br>• Doesn't address annotation provenance or change tracking	N/A (indexing system, not version control)	Demonstrates that performance improvements are possible with GFF; indexing strategies could be adapted for efficient access to version-controlled annotation repositories
**GTF (GFF2)**	Gene annotation (legacy format, predecessor to GFF3)	• Simpler structure than GFF3<br>• Still supported by some legacy tools	• Less flexible than GFF3<br>• Limited feature type support<br>• Shares all coordinate-based versioning problems of GFF3<br>• Being superseded by GFF3 in modern workflows	Very poor: Similar issues to GFF3, but with less structure	Declining; likely to be fully replaced by GFF3 or successor formats; minimal investment warranted

Continued on next page

Table S1 – *Continued from previous page*

Format	Primary Use	Strengths	Limitations	Version Control Compatibility	Future Potential
**GFA**	Graphical assembly representation (preserving structural variation and uncertainty)	• Represents assemblies as graphs (nodes=segments, edges=connections)<br>• Captures repeats, alternative paths, unresolved haplotypes<br>• Changes affect specific graph elements rather than entire contigs<br>• Preserves assembly evidence and structure<br>• Enables programmatic traversal of contigs and haplotype branches	• Metadata sparsely defined<br>• Large assemblies produce files difficult to validate and integrate<br>• Requires substantial auxiliary tooling<br>• Text-based format limits its efficiency for large datasets<br>• Still challenging for current version control methods	Moderate: Graph structure offers opportunities for genomically-aware version control; better than linear formats, but still lacks formal versioning semantics	Promising foundation for version-aware systems; graph structure could be exploited for tracking assembly evolution; requires development of graph-aware diff algorithms
**GFA2**	Extended graphical assembly (multi-alignment, varying detail levels)	• Specifies assembly graphs at varying detail levels<br>• Represents string graphs at any assembly stage<br>• From initial overlaps to final resolved contigs<br>• More comprehensive than GFA	• Increased complexity over GFA<br>• Less widespread adoption than GFA<br>• Shares validation and integration challenges with GFA<br>• Still text-based with efficiency limitations	Moderate: More detailed graph structure could enable finer-grained version tracking, but adds complexity	Could enable detailed assembly provenance tracking; needs integration with version control concepts; potential for capturing assembly decision trees

Continued on next page

Table S1 – *Continued from previous page*

Format	Primary Use	Strengths	Limitations	Version Control Compatibility	Future Potential
**rGFA**	Reference-based pangenome graphs with stable coordinates	• Preserves linear reference genome coordinates within graph structure<br>• Three additional tags per segment indicate origin<br>• Stable coordinate system enables familiar chromosome coordinates<br>• Represents structural variations as graph branches<br>• Balances linear reference compatibility with graph flexibility	• Relatively new format with limited adoption<br>• Requires reference genome as anchor point<br>• Additional complexity over basic GFA<br>• Still lacks formal version tracking mechanisms	Moderate to good: Stable coordinates could facilitate cross-version comparisons; origin tags provide provenance information	Strong potential for version-aware pangenomics; a stable coordinate system could anchor version tracking; integration with annotation versioning needed
**GAF**	Graph alignment (sequence-to-graph alignments)	• Tab-delimited format for graph alignments<br>• Strict superset of PAF format<br>• Uses stable coordinates from rGFA<br>• Enables paths through pangenome graphs	• Depends on underlying graph formats (GFA/rGFA)<br>• Limited adoption compared to traditional alignment formats<br>• No inherent version tracking	Moderate: Could track alignment changes across graph versions if integrated with a version-aware graph system	Could document how read alignments change as pangenome graphs evolve; useful for tracking annotation evidence across versions
**GBZ**	Compressed pangenome graphs (large-scale applications)	• Uses GBWT index for space-efficient path storage<br>• 2.5-3.6x better compression than gzip on GFA files<br>• Human-genome-sized pangenome graphs fit on desktop computers<br>• Fast loading into memory-efficient structures<br>• Burrows-Wheeler transform partitioned between graph nodes	• Binary format reduces human readability<br>• Focused on storage efficiency rather than versioning<br>• Requires specialised tools for access and manipulation<br>• No built-in version tracking mechanisms	Moderate: Efficient storage could enable practical version repositories; compressed representation reduces the storage burden of multiple versions	Excellent candidate for underlying storage in version-controlled systems; compression efficiency enables storing many pangenome versions; needs integration with semantic diff tools

Continued on next page

Format	Primary Use	Strengths	Limitations	Version Control Compatibility	Future Potential
**PAF**	Pairwise read alignment (minimap output format)	• Simple tab-delimited format<br>• Efficient for representing alignments<br>• Widely used with long-read aligners	• Limited to pairwise alignments (no graphs)<br>• No version tracking<br>• Superseded by GAF for graph contexts	Poor: Standard alignment format with no versioning considerations	Limited; likely to remain as legacy format or be replaced by GAF in graph contexts
**VCF**	Variant calling (SNPs, indels, structural variants)	• Standardised format for genetic variation<br>• Supports rich metadata and genotype information<br>• Widely adopted across the genomics community<br>• Can represent complex variants	• Requires reference genome as anchor<br>• No built-in version tracking for the reference itself<br>• Coordinate-based issues when reference changes<br>• Cannot represent graph-based variation effectively	Poor: Reference-dependent coordinates create versioning challenges; no mechanism to track changes in variant calling methods	Could be adapted to document assembly changes as "variants" from previous versions; integration with graph formats (via GAF) could extend utility; needs formal versioning of variant call sets
**BigWig/ BigBed**	Indexed binary formats for genome browser visualisation (signal tracks and features)	• Efficient random access to genomic regions<br>• Dramatically smaller than text equivalents<br>• Optimised for genome browser performance<br>• BigWig for continuous signal, BigBed for discrete features<br>• R-tree indexing enables fast retrieval	• Binary format reduces human readability<br>• Coordinate-based: tied to specific assembly<br>• No built-in version tracking<br>• Must regenerate files for new assemblies<br>• Proprietary to UCSC, though widely adopted	Poor: Coordinate dependency requires regeneration for assembly updates; no mechanism to track how signals change across versions	Excellent for visualisation but needs integration with assembly versioning; liftover tools can convert between assemblies but lose provenance; could serve as export format from version-aware systems
**WIG/ bedGraph**	Genome-wide continuous signal data (ChIP-seq, RNA-seq coverage)	• Simple text formats for signal tracks<br>• Human-readable<br>• Flexible for various data densities<br>• WIG supports variable/fixed step<br>• bedGraph provides fine-grained control	• Inefficient for large datasets (superseded by BigWig)<br>• Coordinate-based, assembly-specific<br>• No metadata standards<br>• No compression<br>• Large file sizes	Very poor: Text-based inefficiency plus coordinate dependency; being replaced by BigWig in practice	Declining; legacy format maintained for compatibility; users should migrate to BigWig for performance; same assembly versioning challenges

Continued on next page

Table S1 – *Continued from previous page*

**Format**	**Primary Use**	**Strengths**	**Limitations**	**Version Control Compatibility**	**Future Potential**
**BED**	Genomic coordinates and features (browser tracks, regulatory regions, peaks)	• Extremely simple tab-delimited format<br>• Flexible columns (3–12+ fields)<br>• Universally supported by genome browsers<br>• Efficient for coordinate-based operations<br>• Human-readable and easily parsed<br>• Standard for peak calling, ChIP-seq, ATAC-seq results	• Coordinate-dependent: breaks when assemblies update<br>• No standardised metadata beyond basic columns<br>• No feature relationships or hierarchies<br>• Cannot represent uncertainty or alternatives<br>• Optional columns poorly standardised across tools<br>• All limitations of coordinate-based formats for version control	Very poor: Coordinate shifts cause all lines to change; no mechanism to track feature evolution or distinguish coordinate updates from biological changes	Limited as primary format; useful as export from version-aware systems; simplicity ensures continued use for genome browser visualization; could benefit from stable coordinate systems like rGFA
**MetaGraph**	Petabase-scale sequence search (annotated de Bruijn graphs)	• Indexed 67 petabase pairs of sequence data<br>• Entire SRA made searchable<br>• Compression up to 7,416 bp/byte for redundant datasets<br>• $100 for small queries, $0.74/Mbp for large queries<br>• k-mer matching and sequence-to-graph alignment	• Focused on search, not sequence representation<br>• Specialised data structures require expertise<br>• No inherent version tracking<br>• Read-centric rather than assembly-centric	N/A (search infrastructure, not version control)	Proves succinct data structures can handle petabase-scale genomics; compression and indexing techniques could be adapted for version-controlled repositories storing millions of genomes

Continued on next page

Table S1 – *Continued from previous page*

Format	Primary Use	Strengths	Limitations	Version Control Compatibility	Future Potential
**GenBank**	Sequence records with rich annotations (NCBI database format)	• Rich metadata in COMMENT sections<br>• Documents pipeline version and annotation method<br>• Standardized record structure<br>• Includes literature references and feature annotations	• High-level metadata only, not feature-level changes<br>• No machine-readable change documentation<br>• When PGAP updates, no programmatic way to identify changed genes<br>• Version information scattered and unstructured	Poor: Documents what version was used, but not what changed; wholesale re-annotation creates entirely new records with no diff	Metadata structure could be extended for formal version tracking; COMMENT sections could be formalised into machine-readable provenance fields; needs integration with systematic change documentation
